# Curcumin Enhances Cytotoxic Effects of Bortezomib in Human Multiple Myeloma H929 Cells: Potential Roles of NF-κB/JNK

**DOI:** 10.3390/ijms13044831

**Published:** 2012-04-16

**Authors:** Qing-Xian Bai, Xiao-Yan Zhang

**Affiliations:** Department of Hematology, State Center of Bone Marrow Transplantation, Xijing Hospital, Fourth Military Medical University, Xi’an 710032, China; E-Mail: zhishui2004@163.com

**Keywords:** curcumin, PS-341, multiple myeloma, NF-κB, JNK

## Abstract

Combined curcumin and PS-341 treatment has been reported to enhance cytotoxicity and minimize adverse effects through ERK and p38MAPK mechanisms in human multiple myeloma cells. However, whether JNK plays similar role in this process remains unclear. In the present study, we found combined treatment altered NF-κB p65 expressions and distributions in multiple myeloma H929 cells. Western blot analysis showed combined treatment inactivated NF-κB while activated JNK signaling. Pre-treatment with JNK inhibitor SP600125 could attenuate NF-κB inactivation and restored H929 cells’ survival. These results suggested that curcumin might enhance the cytotoxicity of PS-341 by interacting with NF-κB, at least in part, through JNK mechanism.

## 1. Introduction

Multiple myeloma is a kind of plasma cell-derived human cancer that causes multiple bone lesions and disturbs the production of normal blood cells. It is the second most prevalent hematologic malignancy after non-Hodgkin’s lymphoma [1]. Complications of untreated or poorly controlled multiple myeloma can be serious and it is generally thought to be an incurable disease, but remissions may be induced with steroids, chemotherapy, radiotherapy, and stem cell transplants. Bortezomib, (PS-341, Velcade^®^) specifically targeting the ubiquitin-proteasome pathway, is the first defined therapeutic proteasome inhibitor approved by FDA for treating refractory, advanced or rapidly relapsed multiple myeloma [2]. It is well documented the mechanism of action for PS-341 lies in its highly affinity and specificity to the catalytic site of the 26S proteasome. There is also increasing evidence that the lethal actions of PS-341 may be related to inactivation of NF-κB pathway [3–5]. PS-341 prevents the cleavage of the inhibitors of NF-κBs (IκBs) proteins and interrupts NF-κB translocation from the cytoplasm to the nucleus, therefore inhibiting proliferation and inducing apoptosis [6]. Although PS-341 has been recommended as the first-line therapy for multiple myeloma, the single-agent activity of PS-341 has been limited [7,8]. The PS-341-based clinical therapeutic strategy for multiple myeloma usually includes other chemotherapeutics, such as adriamycin, decaspray, and thalidomide.

Curcumin is a major active ingredient derived from the plant *Curcuma longa* L. Extensive studies during the past two decades have well documented a wide range of biological activities of curcumin, including anti-cancer properties. Epidemiological evidence also suggests that people who eat curcumin-rich diets have a lower incidence of human cancers. It is noted that curcumin induces down-regulation of NF-κB through suppression of IκBs, leading to cancer cell apoptosis [9–11]. Meanwhile, the chemosensitizing effect of curcumin has been reported in cancers of the breast, colon, pancreas, gut, liver, lung, prostate, brain, and in multiple myeloma, lymphoma and leukemia [12,13]. Interestingly, it is noted that curcumin shows a synergistic effect when combined with PS-341. We and others have recently reported that curcumin enhances the cytotoxic effect of PS-341 in human multiple myeloma cells through regulating NF-κB and Bcl-2 family proteins expressions [14–17]. These beneficial merits develop curcumin as a potential adjuvant agent to standard chemotherapy, in particular for highly malignant, refractory, and relapsed cancers.

Mitrogen-activated protein kinases (MAPKs) family consists of extracellular signal-regulated kinase (ERK), p38MAPK, and c-Jun NH_2_-terminal kinase (JNK), which participated in numerous physiological processes. Expanding studies indicate that ERK and p38MAPK signalings are implicated in manipulating NF-κB and its downstream targets as a response to curcumin in human multiple myeloma cells. However, the distinct roles of JNK in this process remain to be investigated [15,18,19]. To address these questions, we therefore explored potential roles of JNK in curcumin-mediated NF-κB signal in human multiple myeloma H929 cells.

## 2. Results and Discussion

### 2.1. Curcumin and PS-341 Altered Expression and Distribution Profiles of NF-κB p65

**Figure 1:**
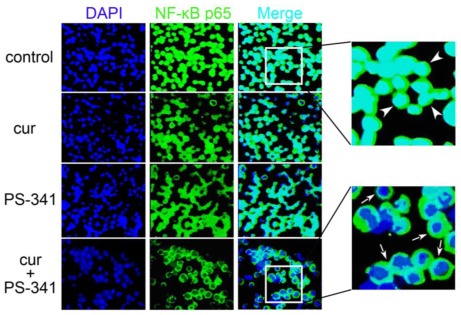
Immunoflurencent staining showing the expression and distribution of NF-κB p65 in response to curcumin with or without PS-341. Cell nucleus were stained with DAPI and visualized by blue fluorescence. NF-κB p65 was stained with corresponding antibodies and visualized by green fluorescence. The distribution of NF-κB p65 within the cell nucleus was indicated by the arrow heads and arrows in the enlarged version.

NF-κB is a dimer mainly composed of NF-κB p65 and NF-κB p50 subunits. Upon activation, NF-κB p65 was released from the NF-κB transcription complex, bound to corresponding DNA and regulated the transcription of specific genes. Therefore, NF-κB p65 is an inducible and functional subunit of NF-κB transcription complex, which provides the gene’s regulatory activities. NF-κB p65 in H929 cells receiving various treatments was visualized by immunoflurencent staining (Figure 1). We observed that numerous H929 cells were NF-κB p65 positive and found that NF-κB p65 was distributed in the whole nucleus (as the arrow head indicated). The proportions of NF-κB p65-expressing cells seemed to decrease in H929 cells supplemented with curcumin or PS-341. Combined curcumin and PS-341 treatment notably decreased the amount of NF-κB p65 positive H929 cells and NF-κB p65 green fluorescent density. Notably, NF-κB p65 mainly distributed in the periphery of the nucleus after combined treatment (as the arrow indicated).

### 2.2. Curcumin and PS-341 Stabilized IκB through JNK Mechanism

**Figure 2:**
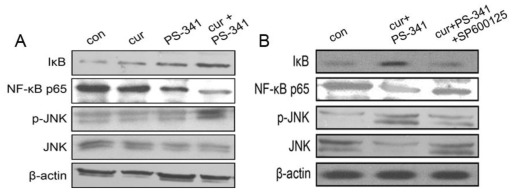
Western blot analysis of IκB, NF-κB p65, p-JNK and JNK. (**A**) H929 cells received curcumin with or without PS-341 treatments. Combined treatment significantly activated JNK signaling and inhibited NF-κB activity; (**B**) Pretreatment with JNK inhibitor SP600125 inhibits JNK phosphorylation, and abolished effects of combined treatment on NF-κB activity. β-actin was used for equal loading.

As the expressions and activities of NF-κB p65 are mainly regulated by IκB, Western blot was employed to analyze the content of IκB and NF-κB p65 after the indicated treatments. Incubation with curcumin or PS-341 for 24 h stabilized IκB and decreased NF-κB p65 content in H929 cells (Figure 2A). Combined treatment exerted a remarkable effect on the stabilization of IκB, which in turn inhibited the expression and transcription activities of NF-κB p65.

ERK and p38MAPK have been reported to contribute to curcumin- and PS-341-mediated IκB stabilization and NF-κB expressions in human multiple myeloma cells *in vivo* and *in vitro*. Blockage of ERK and p38MAPK signaling restored NF-κB activation and promoted IκB degradation [15,18,19]. However, whether or not the JNK mechanism plays similar roles in the presence of curcumin and PS-341 is still unclear. We therefore investigated JNK expressions after the indicated treatment. As the activation of JNK requires phosphorylation (p-JNK), it was observed that treatment with curcumin or PS-341 singularly did not significantly affect JNK phosphorylation. Similarly to previous studies, combined curcumin and PS-341 treatment markedly promoted the expression of p-JNK compared with the baseline (Figure 2A). However, pre-treatment with the JNK specific inhibitor SP600125 attenuated NF-κB p65 inactivation and JNK phosphorylation (Figure 2B). Herein we raise the hypothesis that the mechanism of action for curcumin in combination with PS-341 treatment on NF-κB signaling might be JNK-dependent.

### 2.3. Blockage of JNK Restored H929 Cells Survival

**Figure 3:**
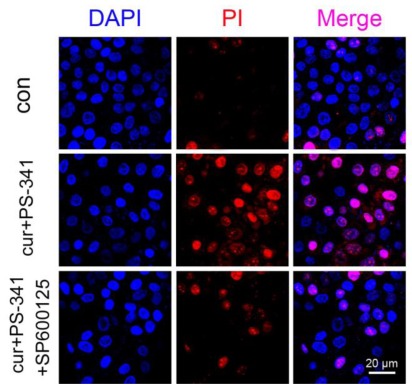
DAPI and PI double staining of H929 cells. Cell nucleus was visualized by DAPI. Cells undergoing necrosis were stained by PI. Cells were observed at ×200 magnification, scale bar = 20 μm.

In order to test this idea, DAPI and PI fluorescent stainings were performed to determine cell viability and apoptosis. The blue fluorescence of DAPI represents the cell nucleus. Because PI cannot penetrate the livingcells, the PI fluorescence is only observed in the necrotic and dead cells. There were few PI positive H929 cells without curcumin and PS-341 treatment (Figure 3). However, combined curcumin and PS-341 treatment provoked remarkable H929 cell death compared with the baseline. As expected, pre-treatment with SP600125 decreased the numbers of PI positive cells. These results indicated that the JNK inhibitor SP600125 attenuated the cytotoxicity of combined treatment.

**Figure 4:**
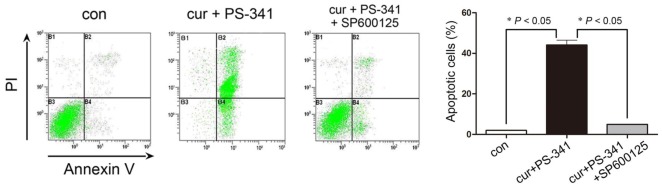
Flow cytometry analysis of cell apoptosis with or without SP600125 pre-treatment. * *P* < 0.05 *vs.* control or SP600125 pretreatment.

We next evaluated pre-treatment with SP600125 on H929 cells apoptosis by flow cytometry. Less than 2% of cells underwent apoptosis without any treatments. Combined curcumin and PS-341 treatment led to profound apoptotic cell death compared with the baseline. However, pre-treatment with SP600125 reduced the apoptotic ratio from (40.62 ± 4.619)% to (5.32 ± 1.155)%, suggesting combined treatment on the NF-κB signaling and H929 cells apoptosis was JNK-dependent (Figure 4).

## 3. Experimental Section

### 3.1. Reagents

Medicine: Curcumin was purchased from Sigma (St Louis, MO, USA). PS-341was purchased from Millennium Pharmaceuticals (Cambridge, MA, USA). SP600125 was purchased from Biomol Research Laboratories (Plymouth Meeting, PA). Curcumin, PS-341 and SP600125 were dissolved in DMSO (1 mM) as stock solutions, stored at −20 °C, and diluted to indicate concentration with culture medium.

Materials: Penicillin, streptomycin, fetal bovine serum and RPMI 1640 were purchased from Invitrogen (Carlsbad, CA, USA). Antibodies against IκB, NF-κB p65, JNK, p-JNK were purchased from Santa Cruz (Santa Cruz, CA, USA). Rabbit anti-β-actin antibody was purchased from Abcam (Cambridge, MA, USA).

### 3.2. Cell Culture

Human multiple myeloma H929 cells were obtained from American Type Culture Collection, and cultured in RPMI 1640 medium supplemented with 10% FBS, penicillin (100 U/mL) and streptomycin (100 μg/mL) in an atmosphere with 5% CO_2_ at 37 °C. In all experiments, exponentially growing cells were used.

### 3.3. Fluorescent Immunocytochemistry of NF-κB p65

After indicated treatments, cells were fixed with 4% paraformaldehyde in PBS for 20 min and permeabilized with 0.5% Trixon X-100 for 15 min. To identify the cell nucleus, cells were incubated with 10 μL DAPI. NF-κB p65 was visualized by incubation with rabbit anti-NF-κB p65 antibody followed by corresponding FITC-labeled secondary antibody. Cells were then viewed and photographed. DAPI and FITC fluorescence were immediately observed under a fluorescent microscope (Olympus, Japan).

### 3.4. Assay of IκB, NF-κB p65 and JNK

IκB, NF-κB p65, JNK and p-JNK protein contents in response to various treatments were determined by Western blot as described previously [14–17].

### 3.5. Analysis of JNK in H929 Cell Apoptosis

To investigate distinct roles of JNK in combined treatments mediated H929 cell apoptosis, SP600125, a specific JNK inhibitor was used to block the phosphorous and activity of JNK. Cells were pre-treated with 5 μM SP600125 (SP600125 was not cytotoxic below 10 μM) for 1 h followed by combined curcumin and PS-341 treatment. JNK, p-JNK, IκB and NF-κB p65 protein contents were determined by Western blot as have described. For cell viability assay, H929 cells were stained by DAPI and PI. The necrotic and dead H929 cells were observed at ×200 magnification under a fluorescent microscope (Olympus, Japan). Quantitatively analysis of cell apoptosis was determined by flow cytometry as have described.

### 3.6. Statistical Analysis

Data are expressed as means ± SD. Differences between groups were analyzed by one-way analysis of variance (ANOVA) followed by *LSD* post hoc test using SPSS statistical software (SPSS, Inc., Chicago, IL). Significance was considered at * *P* < 0.05.

## 4. Conclusion

In the present study, we found that curcumin as a chemosensitizer could enhance the cytotoxic effect of PS-341. Combined curcumin and PS-341 treatment interacts with NF-κB signaling and alters NF-κB p65 expressions and distributions. Pre-treatment with JNK inhibitor SP600125 restored H929 cells’ survival and attenuated NF-κB inactivation as a response to curcumin and PS-341. These results suggest the cytotoxic effect of combined treatment on H929 cells attributes to interaction with NF-κB signaling, and this mechanism might be partly JNK-dependent.
